# Supplementary material for: Augmenting zero-Kelvin quantum mechanics with machine learning for the prediction of chemical reactions at high temperatures
Source: Nat Commun. 2021 Dec 1;12:7012. doi: 10.1038/s41467-021-27154-2 (PMC8636515; doi:10.1038/s41467-021-27154-2)
Supplement: Supplementary file 1 — Supplementary Information [file 41467_2021_27154_MOESM1_ESM.pdf]

Supplementary Information

Augmenting zero-Kelvin quantum mechanics with machine learning for the prediction of chemical reactions at high temperatures

J. A. Garrido Torres *et al.*

1 Supplementary methods

Compound representation (fingerprint)

The compound fingerprint that enters our ML model as input includes the following features obtained from compound properties:

1. Average of constituent metal properties: atomic mass, atomic number, oxidation state, electronegativity, volume, density, center of mass, bulk modulus;
2. Oxide properties: atomic mass, volume, density, center of mass, bulk modulus;
3. Difference between average of constituent metal and oxide properties: volume, density, bulk modulus; and
4. Other properties: average ratio of constituent metal species to oxygen, average ionic character of constituent metal-oxygen bonds, formation energy of the oxide.

A textual description of the individual features is given in **Table 3**, and a reference implementation is included among the enclosed Python code.

Feature selection

The features of our ML model were selected using recursive feature elimination (RFE). This means, individual features were removed from the set of features, and the impact on the model accuracy was assessed with LOOCV. **Fig. 2** shows the change of the model accuracy (based on the mean absolute error, MAE) when the models are built excluding specific features with respect to the model built including all of the features. A negative value means that excluding the feature(s) improves the accuracy of the model, i.e., its error decreases. Excluding the space group feature alone has a strong positive impact on the performance of the model and reduces the LOOCV MAE by nearly 20%, indicating that the feature was mostly redundant. Excluding further features along with the space group did generally not further decrease the model error, and as an example excluding the oxidation state feature is shown in the figure. Note that changes in the MAE are not additive (as seen for the space group/oxidation state combination), and it is therefore challenging to determine the absolute best set of features. However, by recursive elimination, our non-exhaustive tests showed excluding the space group feature to be most important.

Generalization ability of the ML model

In the main text and above, we report the performance of our DFT/ML hybrid model using leave-one-out cross-validation (LOOCV), which shows that the model is robust for predictions of unseen oxides. As an additional test to determine whether our models are able to generalize or are overfitted, we also studied the impact of varying the noise parameter ε of the Gaussian Process (GP) on the goodness-of-fit of the models. An abrupt variation in the goodness-of-fit due to a small change in the noise parameter, which controls the bias-variance trade-off, is an indicator for an overfitted GP. We computed the goodness-of-fit (R^2) of the model trained for predicting reduction temperatures (**Fig. 5**) and the model trained on the free-energy change with the temperature (**Fig. 6**) when using the noise parameters between 10^{-4} and 10^{-1}. For both models, the variation of R^2 is only small (2.5% and 2.2%, respectively), even when the noise parameter is varied by four orders of magnitude. This result indicates that it is very unlikely that our models are overfitted.

Model selection

Besides Gaussian process regression, we also explored other regression models, and the results from LOOCV are shown in **Table 5**. All of the models were constructed with scikit-learn[4], and all of the features were standardized before fittings. For reference, the LOOCV root mean squared error (RMSE), mean absolute error (MAE), and maximum error for Gaussian process regression were 87, 73 and 187 K respectively. As seen in the table, with the exception of support vector regression, the various models show overall very similar performance, and Kernel Ridge regression achieves the lowest errors. However, we are particularly interested in more fine-grained uncertainty estimates offered by Gaussian process regression as it allows us to better understand the reduction order of metals, which is an important factor for industrial processes. As such, and considering the small differences in accuracy, we decided to use Gaussian process regression for our production model.

Formation energy calculations with DFT+U

The calculated formation energies for oxides containing the transition metal species V, Cr, Mn, Co, and Ni were obtained from Hubbard-U corrected[5,6] density-functional theory (DFT) calculations with the SCAN exchange-correlation functional[7] and rVV10 dispersion correction[8], as detailed in the methods section of the main manuscript. Metal oxide energies were obtained using SCAN−rVV10+U whilst the energies of the metals and gas phase molecules were computed using SCAN−rVV10 without Hubbard-U correction, mixing DFT and DFT+U values to calculate the formation energies as follows:

$$\Delta H^{\mathrm{DFT}}_{\mathrm{M_xO_y}} = \frac{2}{y} E^{\mathrm{DFT+U}}_{\mathrm{M_xO_y}} - \frac{2x}{y} E^{\mathrm{DFT}}_{\mathrm{M}} - E^{\mathrm{DFT}}_{\mathrm{O_2}} \quad , \tag{1}$$

where $E^{\mathrm{DFT+U}}_{\mathrm{M_xO_y}}$ is the calculated energy for a given metal oxide using DFT+U whilst $E^{\mathrm{DFT}}_{\mathrm{M}}$ and $E^{\mathrm{DFT}}_{\mathrm{O_2}}$ are the DFT energies computed for the bare metal systems and the oxygen molecule in the gas phase.

To guarantee compatibility of the DFT+U and DFT calculations, the Hubbard-U parameters for the d bands of the transition metals were fitted to the experimental formation energy values from the NIST-JANAF[3] and Cambridge DoITPoMs[1,2] thermodynamic tables (**Table S1**). The U values for those metals presenting more than one oxidation state were fitted to minimize the average error in the formation energies of a set of oxides.

The bar plots in **Fig. 7a,b** show the difference between the experimental and calculated formation energy values when applying different U values for vanadium (**Fig. 7a**) and manganese (**Fig. 7b**). The corresponding average errors are plotted in **Fig. 7c,d**. The optimized U parameters for V and Mn, $U_{\mathrm{V}}(opt.) = 0.7$ eV and $U_{\mathrm{Mn}}(opt.) = 0.5$ eV, were determined by minimizing the absolute average error between the experimental and calculated formation energies. The U values for the other transition metal species (1.5 eV for Cr, 0.3 eV for Co, and 1.5 eV for Ni) were determined equivalently.

2 Supplementary Tables

Supplementary Table 1 Experimental reference data for the different metal oxides. Experimental reduction temperature (T_{red}), standard enthalpy of formation ($\Delta H_{\mathrm{f}}^{0}$), and the change of the formation free-energy with respect to the temperature ($\partial \Delta_f G / \partial T$) for different binary and ternary metal oxides collected from the Cambridge DoITPoMs [1,2] and NIST-JANAF[3] databases.

Compound	T_{red} (K)	$\Delta H_{\mathrm{f}}^{0}$ (kJ mol^{-1})	$\partial \Delta_f G / \partial T$ (kJ mol^{-1} K^{-1})	Compound	T_{red} (K)	$\Delta H_{\mathrm{f}}^{0}$ (kJ mol^{-1})	$\partial \Delta_f G / \partial T$ (kJ mol^{-1} K^{-1})
Li_2O^a	2201	-1205	0.27	$Na_2SiO_3{}^b$	2109	-1039	0.21
Na_2O^a	1359	-843	0.28	$SiO_2{}^a$	1921	-907	0.18
K_2O^a	1098	-724	0.28	$TiO_2{}^a$	2016	-941	0.18
Cu_2O^a	359	-337	0.14	$VO_2{}^a$	1436	-706	0.16
MgO^a	2472	-1202	0.22	$ZrO_2{}^a$	2441	-1092	0.18
CaO^a	2669	-1280	0.22	$LiFeO_2{}^b$	1419	-708	0.17
VO^a	1862	-849	0.16	$Li_2SiO_3{}^b$	2289	-1098	0.21
FeO^a	995	-538	0.14	$Li_2TiO_3{}^b$	2341	-1112	0.20
CoO^a	796	-491	0.16	$Na_2Si_2O_5{}^b$	2093	-985	0.19
NiO^a	715	-471	0.17	$LiAlO_2{}^b$	2395	-1192	0.23
CuO^a	235	-305	0.17	$NaAlO_2{}^b$	2293	-1135	0.22
$Mn_3O_4{}^a$	1354	-692	0.17	$V_2O_5{}^a$	1162	-579	0.13
$Fe_3O_4{}^a$	1005	-551	0.15	$Li_2Si_2O_5{}^b$	2155	-1025	0.20
$Al_2O_3{}^a$	2277	-1125	0.22	$MgAl_2O_4{}^b$	2314	-1155	0.23
$Ti_2O_3{}^a$	2248	-1001	0.17	$Mg_2SiO_4{}^b$	2182	-1094	0.22
$V_2O_3{}^a$	1722	-802	0.16	$LiFe_5O_8{}^b$	1048	-585	0.17
$Cr_2O_3{}^a$	1538	-740	0.16	$Al_2SiO_5{}^b$	2230	-1033	0.19
$Mn_2O_3{}^a$	1192	-636	0.17	$MgTi_2O_5{}^b$	2096	-1006	0.20
$Fe_2O_3{}^a$	924	-543	0.17	$MgTiO_3{}^b$	2114	-1055	0.22

[a]Cambridge DoITPoMs [b]NIST-JANAF

Supplementary Table 2 Oxide reduction temperatures. Values of the predicted and reference oxide reduction temperatures are shown in **Figure 1**. The temperatures predicted by density-functional theory without (DFT) and with phonon correction (Phonon) and for the machine-learning (ML) models trained on reduction temperatures (T_{red}) and free-energy slopes ($\partial G/\partial T$) are given. The experimental reference values (Expt.) are identical to those of **Table 1**. All reduction temperatures are in Kelvin.

Compound	Expt.	DFT	Phonon	ML (T_{red})	ML ($\partial G/\partial T$)
Al_2O_3	2277	2064	2092	2459 ± 111	2374 ± 173
CaO	2669	2437	2702	2589 ± 227	2546 ± 162
CoO	796	527	597	593 ± 161	721 ± 32
Cr_2O_3	1538	1135	1229	1280 ± 63	1439 ± 99
Cu_2O	359	203	195	366 ± 283	261 ± 29
CuO	235	286	219	448 ± 220	383 ± 19
Fe_2O_3	924	842	891	1004 ± 78	1111 ± 54
Fe_3O_4	1005	784	–	988 ± 62	1076 ± 100
FeO	995	736	739	1019 ± 102	950 ± 34
K_2O	1098	1177	1130	997 ± 423	1119 ± 75
Li_2O	2201	2228	2179	2171 ± 315	2250 ± 182
MgO	2472	2252	2366	2452 ± 196	2448 ± 168
Mn_2O_3	1192	1008	–	1289 ± 66	1217 ± 84
Mn_3O_4	1354	1059	–	1288 ± 39	1359 ± 60
Na_2O	1359	1436	1392	1479 ± 227	1426 ± 56
NiO	715	588	–	997 ± 306	762 ± 63
Ti_2O_3	2248	1817	–	2401 ± 211	2281 ± 130
V_2O_3	1722	1364	1405	1742 ± 158	1726 ± 71
V_2O_5	1162	864	922	1314 ± 89	1156 ± 120
VO	1862	1425	1461	1648 ± 211	1807 ± 163
VO_2	1436	1157	1212	1292 ± 86	1517 ± 144
ZrO_2	2441	2085	2209	2361 ± 394	2552 ± 273
Al_2SiO_5	2230	1823	–	2130 ± 161	2122 ± 78
$Li_2Si_2O_5$	2155	1787	–	2104 ± 149	2103 ± 130
Li_2SiO_3	2289	1962	–	2255 ± 29	2171 ± 45
Li_2TiO_3	2341	2040	–	2334 ± 157	2300 ± 116
$LiFe_5O_8$	1048	817	–	1136 ± 168	1058 ± 105
$LiFeO_2$	1419	1210	–	1347 ± 111	1404 ± 97
Mg_2SiO_4	2182	1939	–	2240 ± 123	2279 ± 58
$MgTi_2O_5$	2096	1785	–	2116 ± 132	2177 ± 80
$MgTiO_3$	2114	1885	–	2097 ± 56	2205 ± 180
$Na_2Si_2O_5$	2093	1694	–	2015 ± 177	1961 ± 82
Na_2SiO_3	2109	1785	–	2128 ± 160	2084 ± 73

Supplementary Table 3 Description of the features that enter the compound fingerprint.

Feature	Description
Ratio metal oxygen (mean)	Mean ratio of the number of constituent metal atoms to the number of oxygen atoms
Oxidation state (mean)	Composition weighted mean oxidation state of constituent metal atoms
Atomic number (mean)	Mean atomic number of constituent metal atoms
Electronegativity (mean)	Mean electronegativity of constituent metal atoms
Ionic character (mean)	Mean ionic character of constituent metal-oxygen bonds
Volume M (mean)	Mean volume per formula unit of constituent metal atoms
Volume MO	Volume per formula unit of the oxide
Difference Volume (M-MO) (mean)	Difference between two features above
Center of mass M (mean)	Mean center of mass of constituent metal atoms
Center of mass MO	Center of mass of the oxide
Mass M (mean)	Mean atomic mass of constituent metal atoms
Mass of MO	Atomic mass of the oxide
Mass of MO (only metals)	Composition weighted mean atomic mass of constituent metal atoms
Density M (mean)	Mean density of constituent metal atoms
Density MO	Density of the oxide
Difference Density (M-MO) (mean)	Difference between two features above
Bulk modulus M (mean)	Mean bulk modulus of constituent metal atoms
Bulk modulus MO	Bulk modulus of the oxide
Difference Bulk modulus (M-MO) (mean)	Difference between two features above
Formation energy	Formation energy of the oxide in kJ/mol

Supplementary Table 4 Error estimates of the four models discussed in the main text. The mean absolute error (MAE) and the root mean squared error (RMSE) were determined by leave-one-out cross-validation. The error estimates are given both for the entire data set of Table S1 and for a subset containing only the binary oxides for which phonon calculations were performed. The final row shows the error estimates for a baseline model for which the zero-Kelvin formation enthalpies were taken from experiment (Table S1) instead of DFT.

Model	MAE (K) (only binary)	MAE (K) (binary and ternary)	RMSE (K) (only binary)	RMSE (K) (binary and ternary)
DFT	235	256	265	277
DFT+Phonon	166	–	202	–
ML (T_{red})	105	85	127	109
ML ($\partial G/\partial T$)	74	64	87	78
ML ($\partial G/\partial T$, H_f^{expt})	46	53	57	65

Supplementary Table 5 Error estimates for different regression models. The mean absolute error (MAE) and the root mean squared error (RMSE) were determined by leave-one-out cross-validation. The final row shows the maximum error (in reduction temperature) and the corresponding metal oxide.

Regression model	RMSE (K)	MAE (K)	Max. error (K)
Linear	75	61	176, CaO
Ridge	77	62	191, CaO
LASSO	78	61	181, Li_2O
ElasticNet	80	63	223, Li_2O
Support Vector	147	127	376, Li_2O
Kernel Ridge	69	57	165, CaO
Random Forest	93	69	289, Al_2O_3
Gradient Boosting	74	56	250, Li_2O
AdaBoost	91	58	299, CaO
ExtraTrees	75	59	243, CaO

3 Supplementary Figures

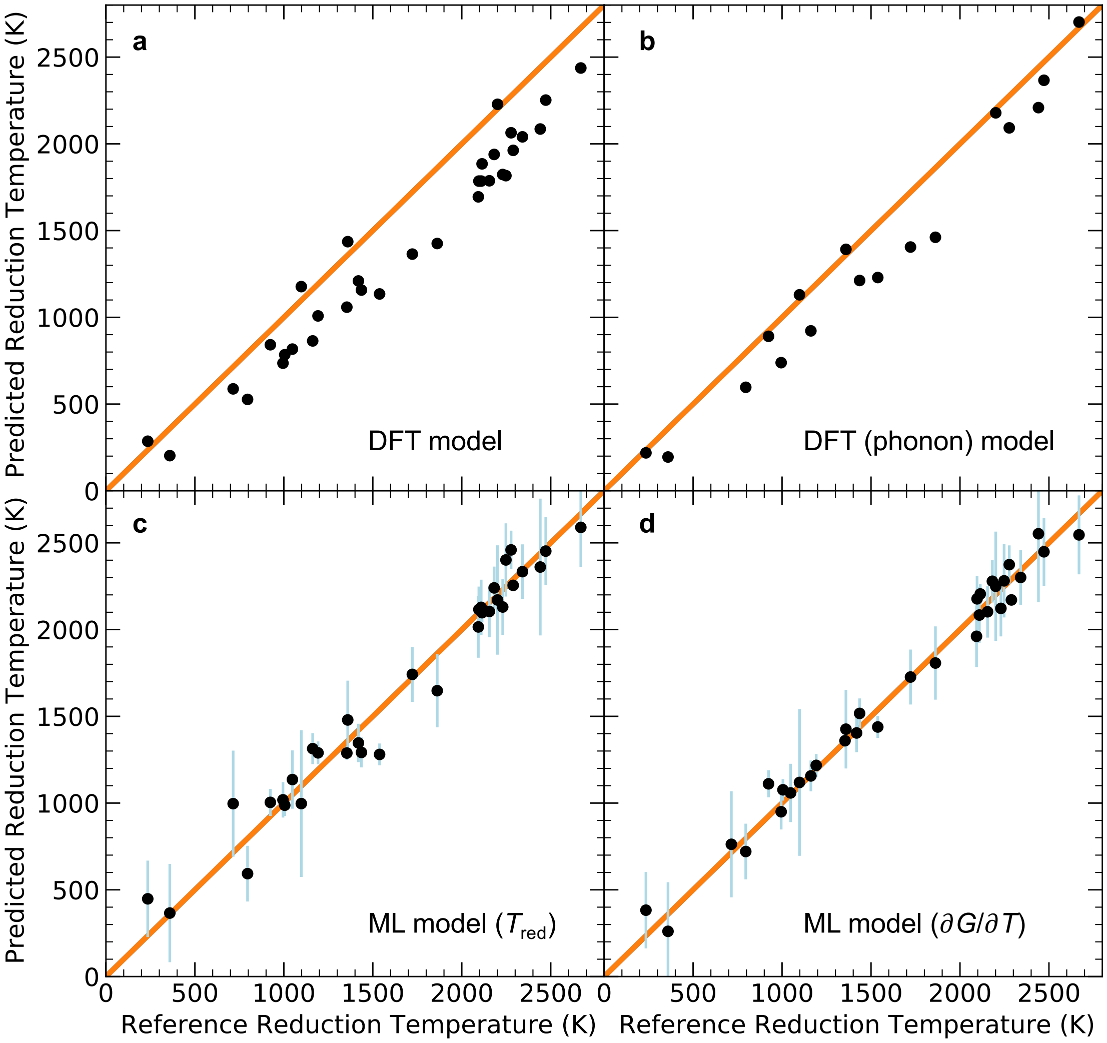

Supplementary Fig. 1 Correlation of predicted and reference reduction temperatures. The oxide reduction temperatures as predicted by **a,** density-functional theory (DFT) only considering the temperature-dependent entropy of CO gas, **b,** DFT including phonon corrections for the vibrational entropy of the solid phases, **c,** the machine learning (ML) model trained on reduction temperatures, and **d,** the ML model trained on free-energy slopes are compared to the experimental reduction temperatures from **Table 1**. The vertical error bars in panels **c** and **d** are the uncertainties predicted by the respective Gaussian process regression models. Panels **c** and **d** show values from leave-one-out cross-validation that were not included in the model construction. The diagonal orange lines correspond to perfect agreement. The data shown in the plots is given also in **Table 2**.

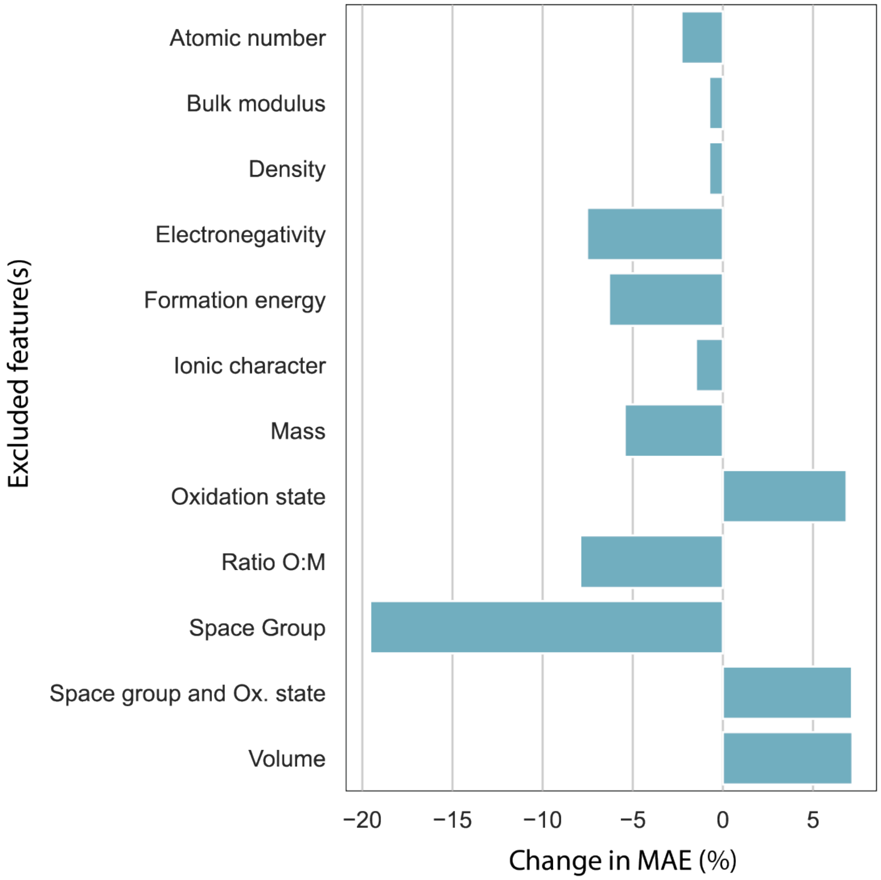

Supplementary Fig. 2 Feature selection. Relative change of the Mean Absolute Error (MAE) when excluding specific features compared to the MAE when all of the features were used.

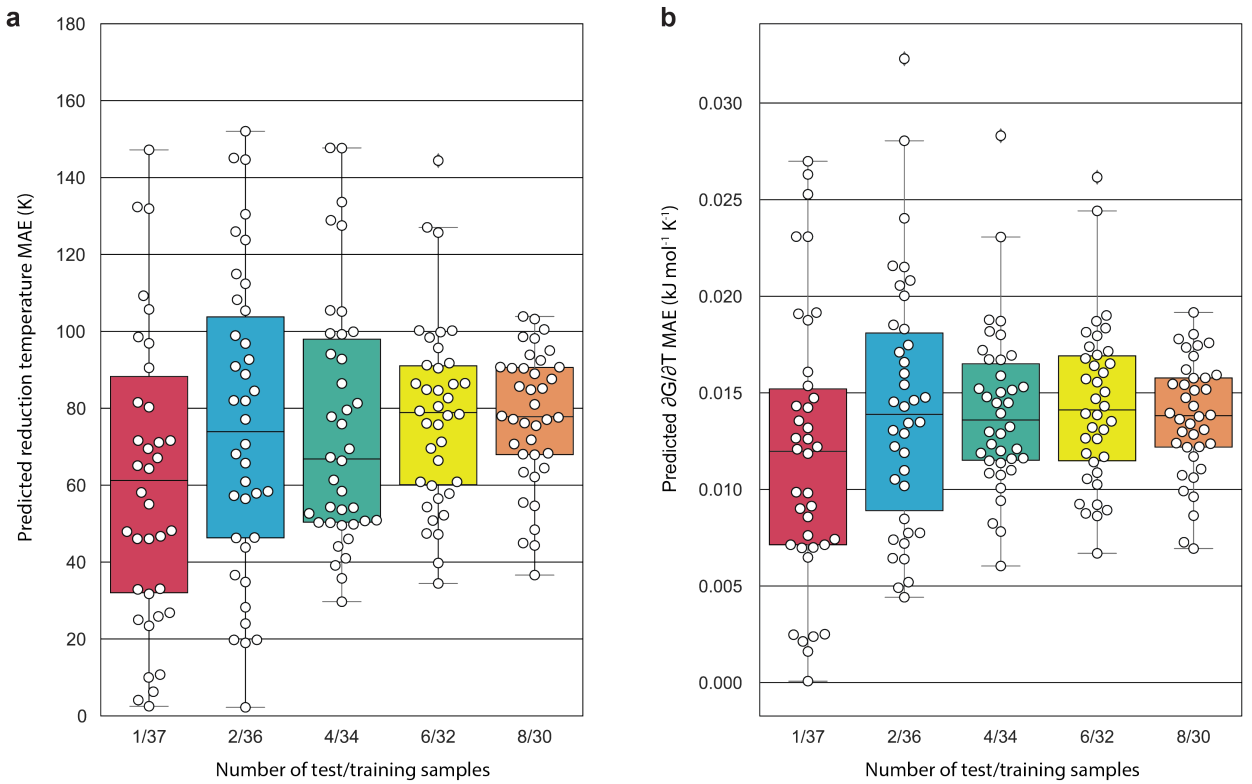

Supplementary Fig. 3 ***k*-fold cross-validation of the machine learning models.** Cross-validation with different test/train splits to evaluate the robustness of the two different machine learning models used for predicting **a**, reduction temperatures and **b**, the oxide formation free-energy change with the temperature. Random samples are drawn to statistically assess the performance of the model when using a given test/train split. The white circles indicate the mean absolute error (MAE) for a given random sample and test/train split. The median MAE in each case is indicated by a horizontal line within the boxes. The box height corresponds to the interquartile range (IQR), and the error bars (whiskers) extend to the extreme points within ±1.5×IQR relative to the ends of the boxes.

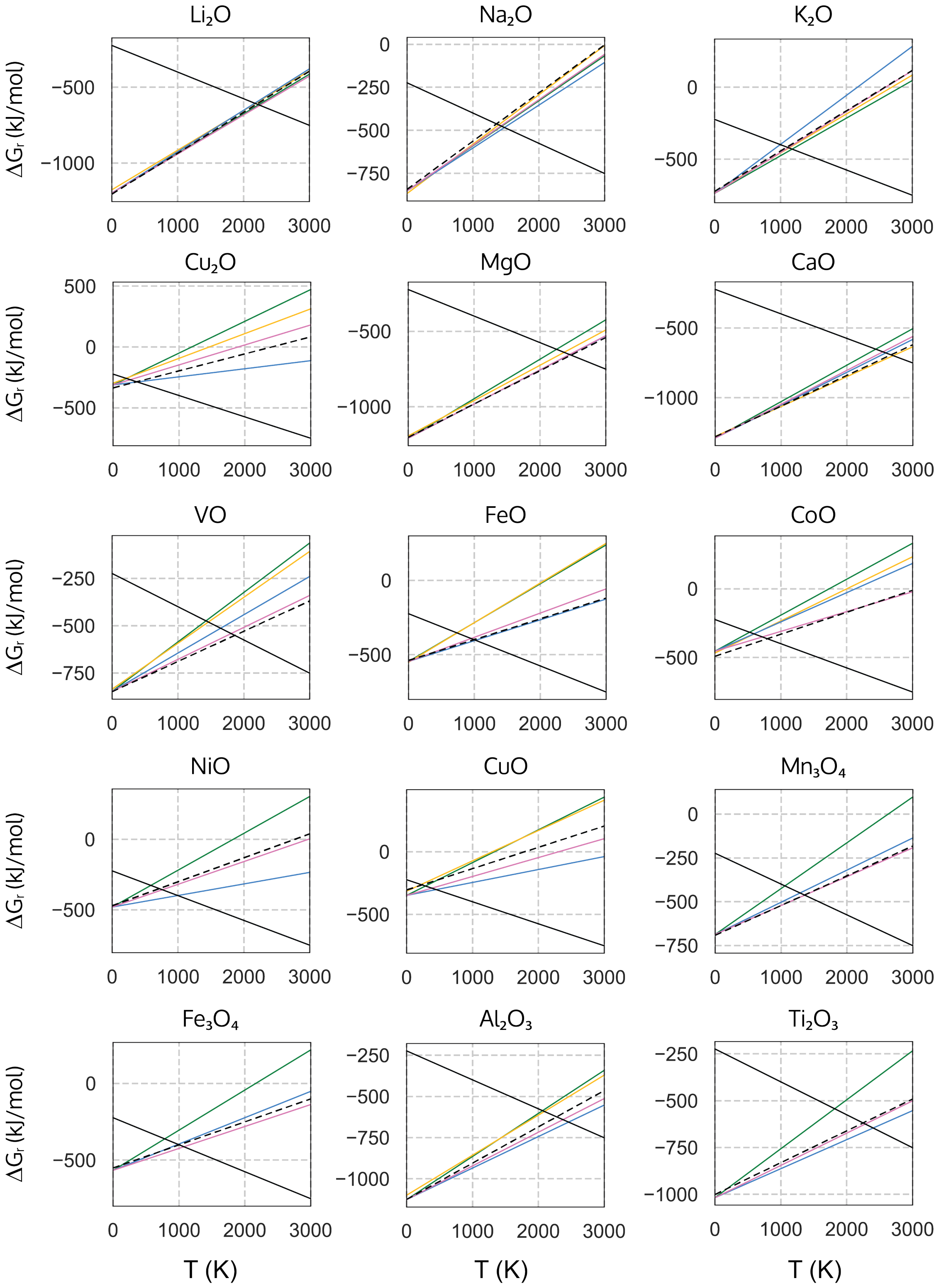

Supplementary Fig. 4 Free-energy diagrams (Part I). Free-energy curves obtained using DFT (green), DFT including phonon corrections (orange) and the ML models trained on reduction temperatures (blue) and free-energy slopes (pink) are compared with their corresponding experimental reference data (black dashed lines). The black solid lines indicate the experimental free-energy curve for CO formation.

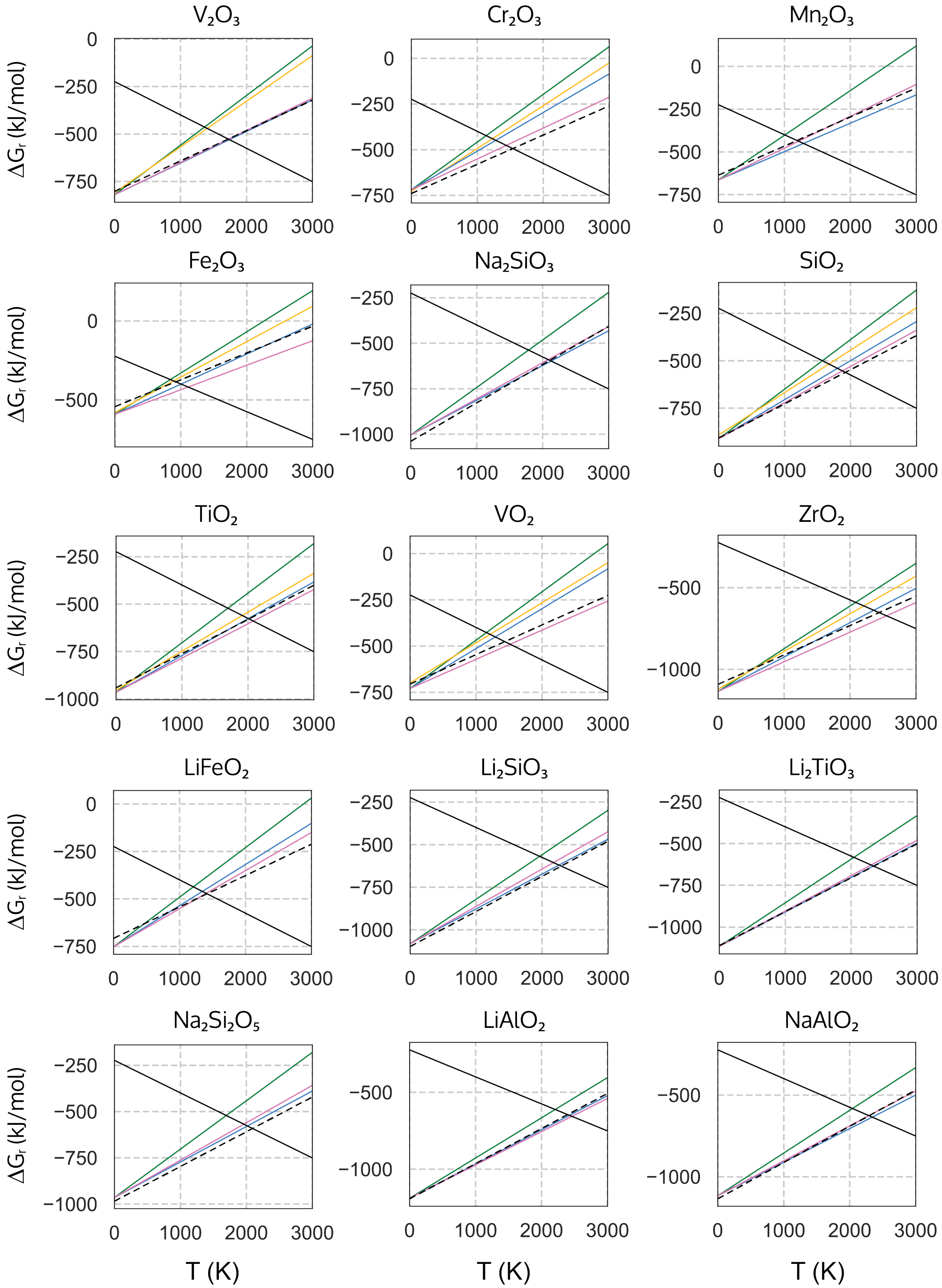

Supplementary Fig. 4 Free-energy diagrams (Part II).

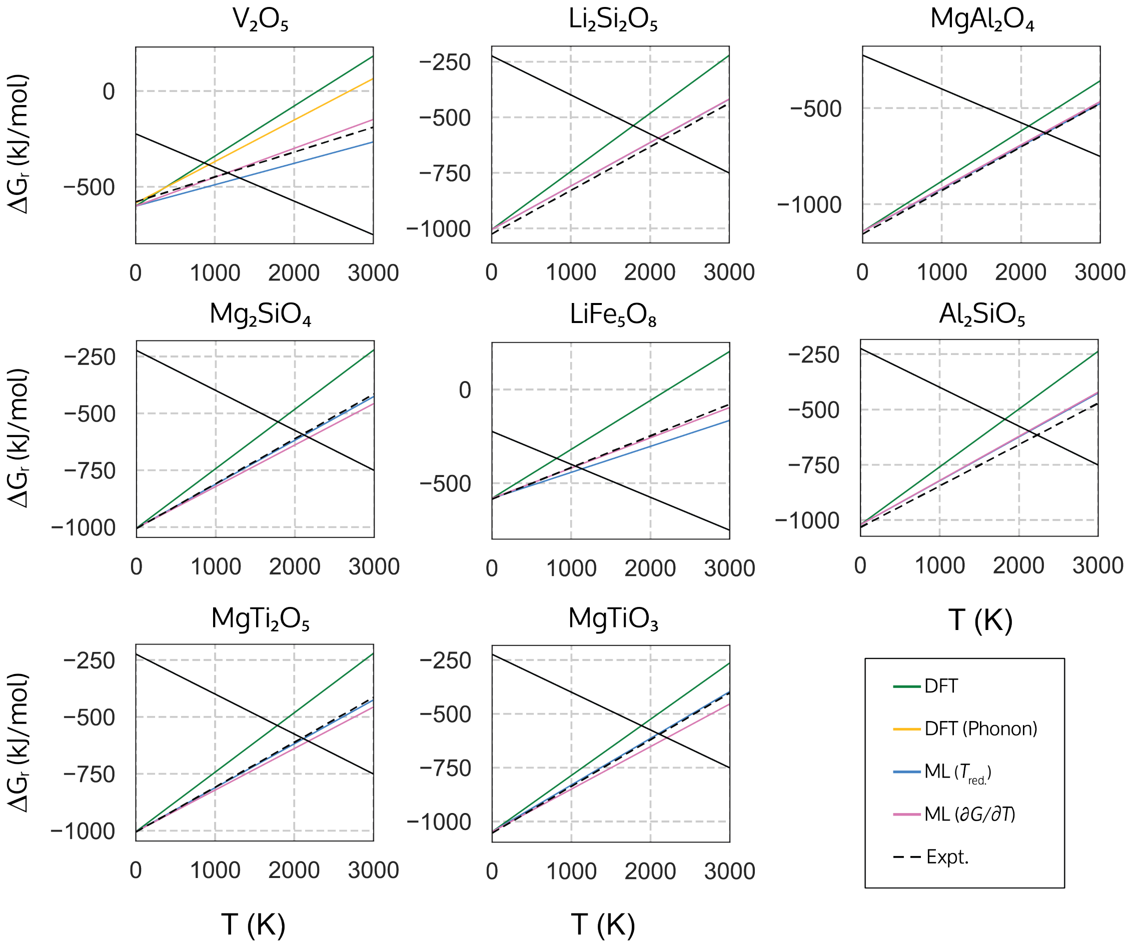

Supplementary Fig. 4 Free-energy diagrams (Part III).

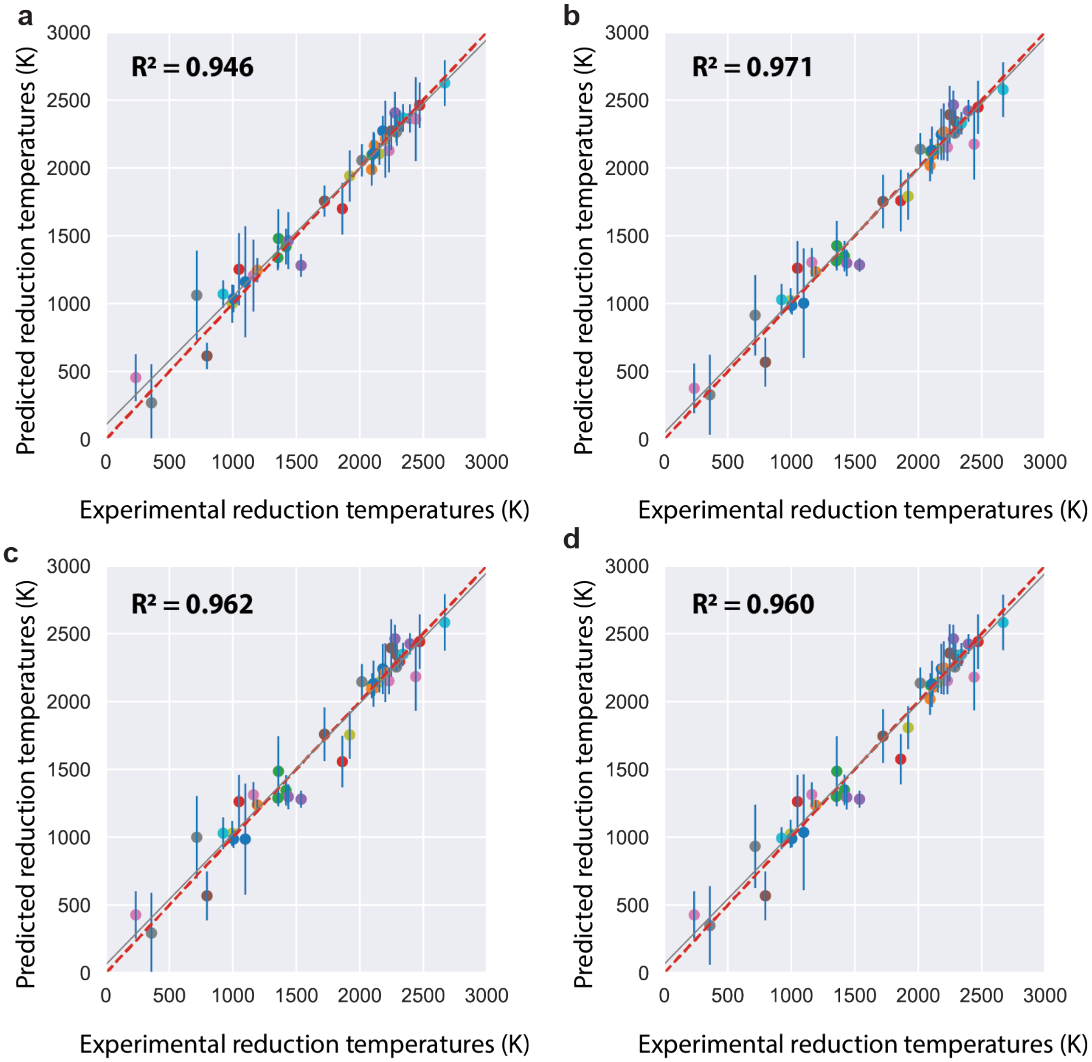

Supplementary Fig. 5 Reduction temperature model accuracy when varying the GP noise parameter. Comparison between experimental and predicted formation free-energy changes when building the Gaussian Process (GP) regression models using the following noise parameters ε: (a) 10^{-1}, (b) 10^{-2}, (c) 10^{-3} and (d) 10^{-4}. Error bars indicate the uncertainty estimate of the GP model.

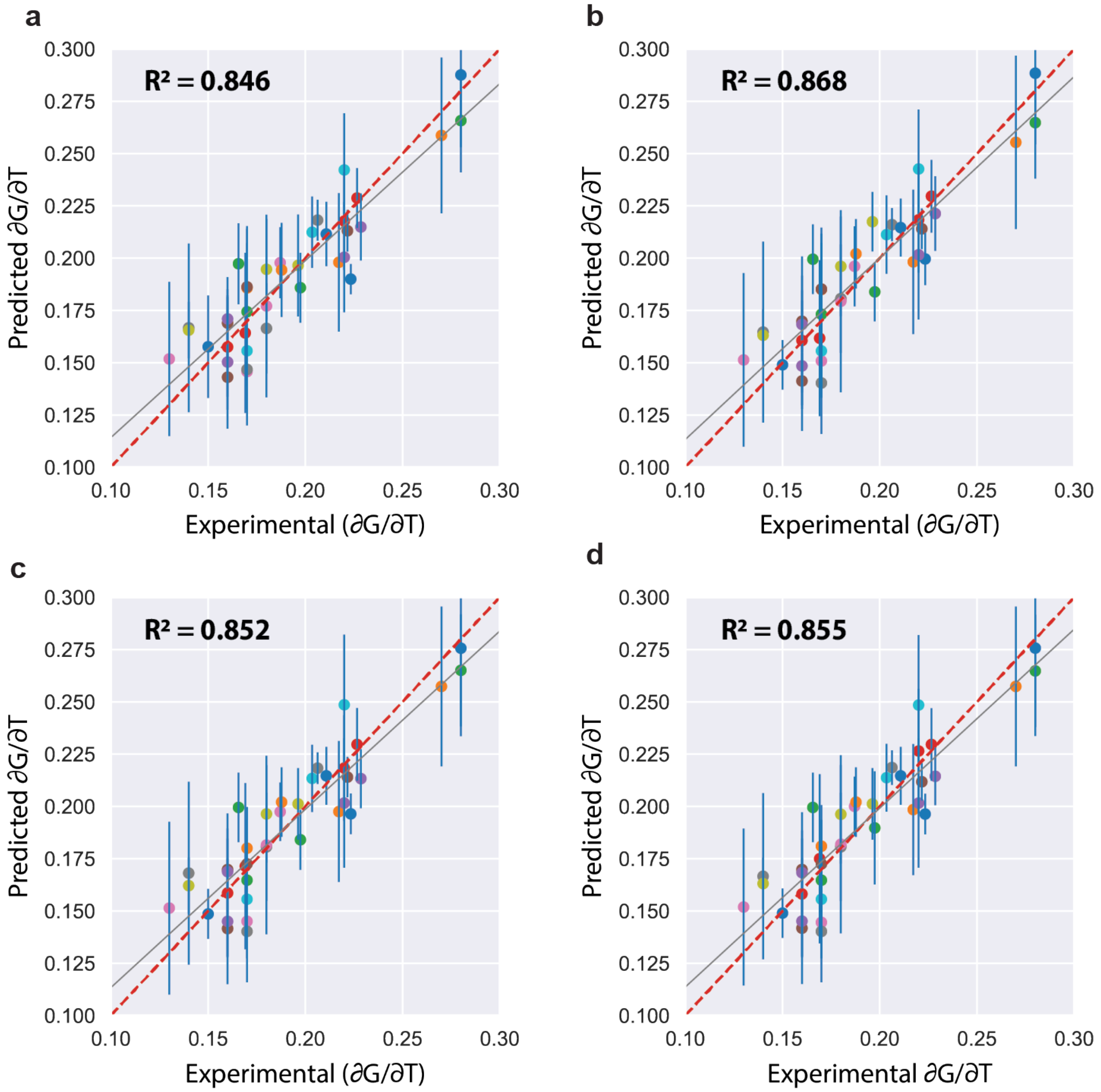

Supplementary Fig. 6 Free-energy slope accuracy when varying the GP noise parameter. Comparison between experimental and predicted formation free-energy changes when building the Gaussian Process (GP) models using the following noise parameters ε: (a) 10^{-1}, (b) 10^{-2}, (c) 10^{-3} and (d) 10^{-4}. Error bars indicate the uncertainty estimate of the GP model.

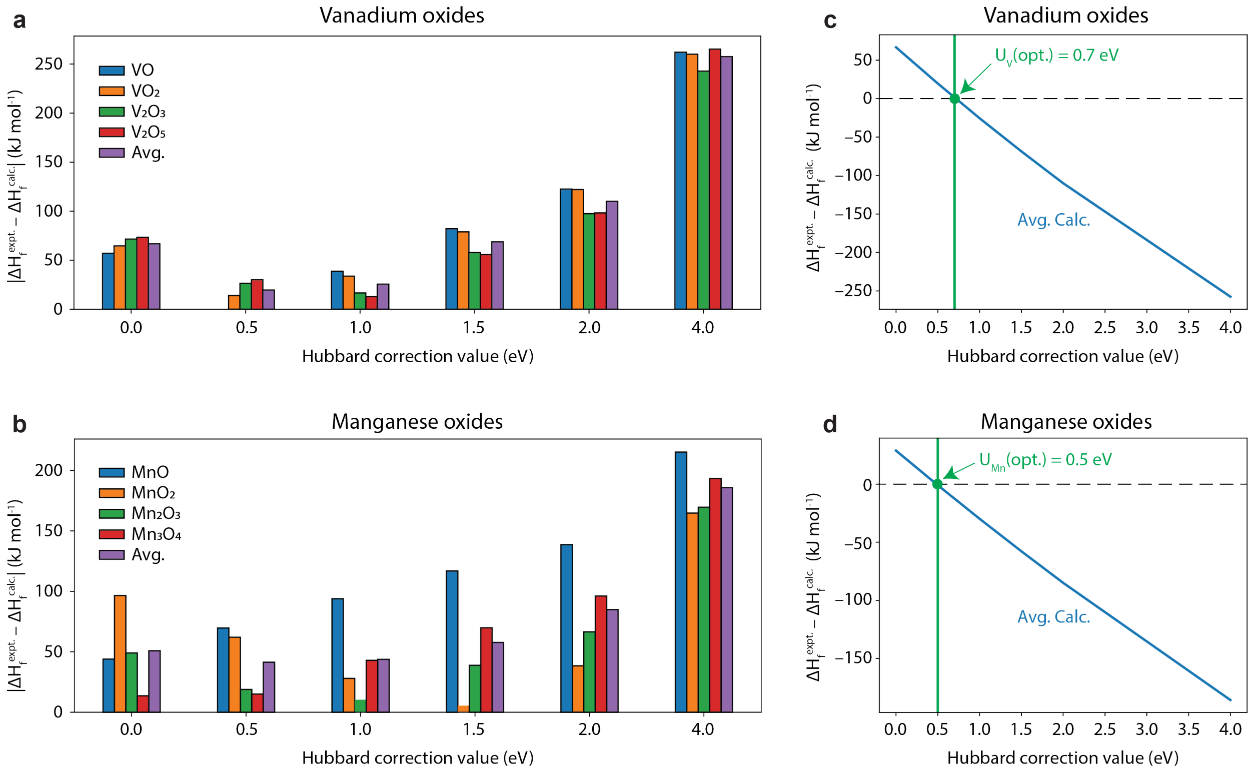

Supplementary Fig. 7 Optimized DFT+U values. a,b, Bar plots representing the absolute error between the experimental and calculated formation energies as a function of the Hubbard U parameter for **a**, V and **b**, Mn. The average errors for the binary oxides of **c**, V and **d**, Mn are represented by the blue solid lines, and the green lines highlight the optimal U values, i.e., the value for which the average error between experiments and theory is zero.

References

[1] Turkdogan, E. Physical chemistry of high temperature technology, new york (1980).

[2] Doitpoms. *Cambridge University* https://www.doitpoms.ac.uk/tlplib (2020).

[3] Chase Jr, M. & Tables, N.-J. T. Forth edition/chase mw, jr. *Journal of Physical and Chemical Reference Data, Monograph* **9**, 1951 (1998).

[4] Pedregosa, F. *et al.* Scikit-learn: Machine learning in Python. *Journal of Machine Learning Research* **12**, 2825–2830 (2011).

[5] Liechtenstein, A., Anisimov, V. I. & Zaanen, J. Density-functional theory and strong interactions: Orbital ordering in mott-hubbard insulators. *Phys. Rev. B* **52**, R5467 (1995).

[6] Dudarev, S., Botton, G., Savrasov, S., Humphreys, C. & Sutton, A. Electron-energy-loss spectra and the structural stability of nickel oxide: An lsda+ u study. *Phys. Rev. B* **57**, 1505 (1998).

[7] Sun, J., Ruzsinszky, A. & Perdew, J. P. Strongly constrained and appropriately normed semilocal density functional. *Phys. Rev. Lett.* **115**, 036402 (2015).

[8] Peng, H., Yang, Z.-H., Perdew, J. P. & Sun, J. Versatile van der waals density functional based on a meta-generalized gradient approximation. *Phys. Rev. X* **6**, 041005 (2016).
