# Supplementary material for: Augmenting zero-Kelvin quantum mechanics with machine learning for the prediction of chemical reactions at high temperatures
Source: Nat Commun. 2021 Dec 1;12:7012. doi: 10.1038/s41467-021-27154-2 (PMC8636515; doi:10.1038/s41467-021-27154-2)
Supplement: Supplementary file 3 — Description of Additional Supplementary Files [file 41467_2021_27154_MOESM3_ESM.pdf]

Description of Additional Supplementary Files

File Name: Supplementary Software 1
Description: Contents of Supplementary Software 1

- data
- experimental_structures
- 1_get_experimental_data.py
- 2_get_ellingham_phonon.py
- 3_machine_learning_red_temperatures.py
- 4_machine_learning_slopes.py
- 5_feature_selection.py
- 6_train_test_split.py
- datagen.py

The directories 'data' and 'experimental_structures' contain experimental reference data in comma-separated values format and crystal structures in CIF format, repsectively. The Python script 'datagen.py' processes these reference data. The remaining Python scripts implement the model construction and validation as described in the manuscript and the supplementary information.
